# Microbial Functional Responses to Cholesterol Catabolism in Denitrifying Sludge

**DOI:** 10.1128/mSystems.00113-18

**Published:** 2018-10-30

**Authors:** Sean Ting-Shyang Wei, Yu-Wei Wu, Tzong-Huei Lee, Yi-Shiang Huang, Cheng-Yu Yang, Yi-Lung Chen, Yin-Ru Chiang

**Affiliations:** a Biodiversity Research Center, Academia Sinica, Taipei, Taiwan; b Graduate Institute of Biomedical Informatics, College of Medical Science and Technology, Taipei Medical University, Taipei, Taiwan; c Institute of Fisheries Science, National Taiwan University, Taipei, Taiwan; University of California, San Diego

**Keywords:** cholesterol, biodegradation, rare biosphere, metatranscriptome, activated sludge, cobalamin, molydoenzyme

## Abstract

The 2,3-*seco* pathway, the pathway for anaerobic cholesterol degradation, has been established in the denitrifying betaproteobacterium Sterolibacterium denitrificans. However, knowledge of how microorganisms respond to cholesterol at the community level is elusive. Here, we applied mesocosm incubation and 16S rRNA sequencing to reveal that, in denitrifying sludge communities, three betaproteobacterial operational taxonomic units (OTUs) with low (94% to 95%) 16S rRNA sequence similarity to Stl. denitrificans are cholesterol degraders and members of the rare biosphere. Metatranscriptomic and metabolite analyses show that these degraders adopt the 2,3-*seco* pathway to sequentially catalyze the side chain and sterane of cholesterol and that two molybdoenzymes—steroid C25 dehydrogenase and 1-testosterone dehydrogenase/hydratase—are crucial for these bioprocesses, respectively. The metatranscriptome further suggests that these betaproteobacterial degraders display chemotaxis and motility toward cholesterol and that FadL-like transporters may be the key components for substrate uptake. Also, these betaproteobacteria are capable of transporting micronutrients and synthesizing cofactors essential for cellular metabolism and cholesterol degradation; however, the required cobalamin is possibly provided by cobalamin-*de novo*-synthesizing gamma-, delta-, and betaproteobacteria via the salvage pathway. Overall, our results indicate that the ability to degrade cholesterol in sludge communities is reserved for certain rare biosphere members and that C25 dehydrogenase can serve as a biomarker for sterol degradation in anoxic environments.

**IMPORTANCE** Steroids are ubiquitous and abundant natural compounds that display recalcitrance. Biodegradation via sludge communities in wastewater treatment plants is the primary removal process for steroids. To date, compared to studies for aerobic steroid degradation, the knowledge of anaerobic degradation of steroids has been based on only a few model organisms. Due to the increase of anthropogenic impacts, steroid inputs may affect microbial diversity and functioning in ecosystems. Here, we first investigated microbial functional responses to cholesterol, the most abundant steroid in sludge, at the community level. Our metagenomic and metatranscriptomic analyses revealed that the capacities for cholesterol approach, uptake, and degradation are unique traits of certain low-abundance betaproteobacteria, indicating the importance of the rare biosphere in bioremediation. Apparent expression of genes involved in cofactor *de novo* synthesis and salvage pathways suggests that these micronutrients play important roles for cholesterol degradation in sludge communities.

## Abstract (precis)

Steroids are ubiquitous and abundant natural compounds that display recalcitrance. Biodegradation via sludge communities in wastewater treatment plants is the primary removal process for steroids. To date, compared to studies for aerobic steroid degradation, the knowledge of anaerobic degradation of steroids has been based on only a few model organisms. Due to the increase of anthropogenic impacts, steroid inputs may affect microbial diversity and functioning in ecosystems. Here, we first investigated microbial functional responses to cholesterol, the most abundant steroid in sludge, at the community level. Our metagenomic and metatranscriptomic analyses revealed that the capacities for cholesterol approach, uptake, and degradation are unique traits of certain low-abundance betaproteobacteria, indicating the importance of the rare biosphere in bioremediation. Apparent expression of genes involved in cofactor *de novo* synthesis and salvage pathways suggests that these micronutrients play important roles for cholesterol degradation in sludge communities.

## INTRODUCTION

Steroids, representing a major class of triterpenoids, are organic compounds characterized by a planar and a relatively rigid carbon skeleton (sterane). The term “steroids” generally refers to sex hormones and to sterols that contain an aliphatic side chain. The latter include cholesterol, phytosterols, and ergosterol, which are fundamental membrane constituents for animals, plants, and fungi, respectively (1). Sterols are very likely the most abundant steroids in environments (2). The molecular structure of sterols is highly reduced, and this makes them feasible carbon and energy sources for microorganisms. However, sterols display recalcitrance due to their low number of functional groups, low solubility in water, and complex spatial conformation (3). Certain aerobic bacteria, including actinobacteria and proteobacteria, are able to utilize steroids as growth substrates by adopting the 9,10-*seco* pathway to mineralize these recalcitrant structures through specific types of oxygenases (2, 4). The catabolic mechanisms and enzymes involved in side chain degradation (5) and the oxygenolytic cleavage of A/B-rings (6–8) in cholesterol have been extensively studied in Mycobacterium tuberculosis. However, the mechanism of steroid C/D-ring degradation has been addressed only recently (9).

**FIG 1:**
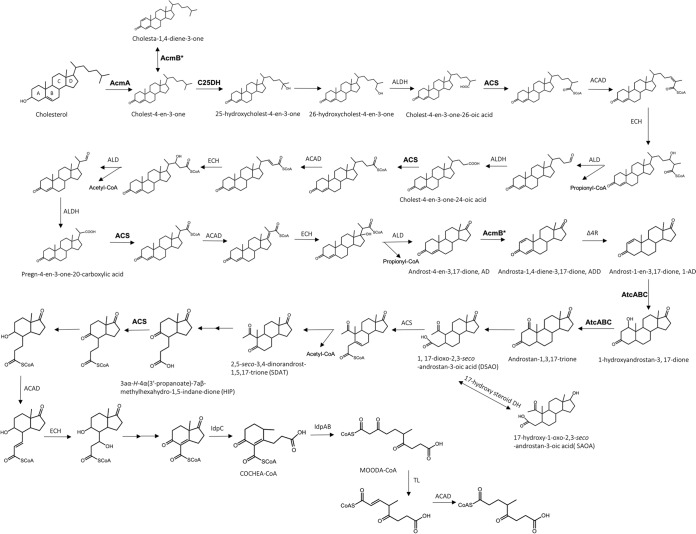
The 2,3-*seco* pathway of anaerobic cholesterol metabolism in Stl. denitrificans. Enzymes functionally characterized in previous studies are bolded. AcmA, cholesterol dehydrogenase/isomerase; AcmB, cholest-4-en-3-one-Δ1-dehydrogenase; C25DH, cholesterol C25 dehydrogenase; ACS, acyl-CoA synthetase; ACAD, acyl-CoA dehydrogenase; ECH, enoyl-CoA hydratase; ALD, aldolase; ALDH, aldehyde dehydrogenase; Δ4R, ketosteroid-Δ4-reductase; DH, dehydrogenase; AtcABC, 1-testosterone dehydrogenase/hydratase.

Knowledge about steroid-degrading anaerobes and their degradation mechanisms remains comparatively limited. A motile betaproteobacterium, Sterolibacterium denitrificans DSM 13999 (Stl. denitrificans), isolated from anoxic sludge of a wastewater treatment plant, was able to use nitrates as terminal electron acceptors to mineralize cholesterol (10). The 2,3-*seco* pathway, an anaerobic cholesterol catabolic pathway, has been established using Stl. denitrificans as a model organism (11, 12), and this anaerobic bioprocess sequentially includes substrate uptake, sterane modification, side chain degradation, A/B-ring degradation, and C/D-ring degradation (Fig. 1). A cholesterol-specific outer membrane protein, the FadL-like transporter, appears to mediate cholesterol uptake across the outer membrane through facilitated diffusion (13, 14). After being transported into the periplasm, the hydroxyl group of ring A is oxidized and the carbon-carbon double bond is isomerized by cholesterol dehydrogenase/isomerase AcmA, yielding cholest-4-en-3-one (13, 15). The anaerobic hydroxylation at C-25 of cholest-4-en-3-one is then catalyzed by the molybdopterin-containing steroid C25 dehydrogenase (C25DH) (comprising α, β, and γ subunits), resulting in 25-hydroxycholest-4-en-3-one (16, 17). Followed by the isomerization of the tertiary hydroxyl group, 26-hydroxycholest-4-en-3-one is produced and its alcohol is oxidized, generating cholest-4-en-3-one-26-oic acid. Subsequently, the activated side chain is degraded through three cycles of β-oxidation reactions to produce the androgen metabolite androst-4-en-3,17-dione (AD) (12). Recently, the coenzyme A (CoA)-ester metabolites and genes involved in this anaerobic side chain degradation have been identified (14), and the substrate specificity of acyl-CoA synthetase required for side chain activation prior to each β-oxidation has also been functionally characterized (18). Most metabolites used in the cholesterol degradation possess corresponding 1,4-diene structures (12, 14), which are transformed by a 3-ketosteroid Δ^1^-dehydrogenase AcmB (15).

The degradation of the cholesterol core-ring structure (sterane) starts from the activation and the following hydrolytic cleavage of the A-ring. After the AD is transformed into androst-1-en-3,17-dione (1-AD) through two sequential redox reactions at C-1 and C-4, a water molecule is introduced to the double bond between C-1 and C-2 of 1-AD by a bifunctional molybdopterin-containing hydratase/dehydrogenase AtcABC (19), producing androstan-1,3,17-trione. This 1,3-dione structure is then hydrolytically cleaved, resulting in a characteristic intermediate, 1,17-dioxo-2,3-*seco*-androstan-3-oic acid (DSAO), for the 2,3-*seco* pathway (12, 19). All of these androgen metabolites possess the corresponding 17-hydroxyl structures (12), which are transformed by a 17-hydroxysteroid dehydrogenase (19). DSAO is then activated by an unidentified acyl-CoA synthase, generating DASO-CoA, and its acetyl-CoA at C-5 is proposed to be degraded by an aldolytic reaction to produce 2,5-*seco*-3,4-dinorandrost-1,5,17-trione (2,5-SDAT) (11). The processes of hydrolytic cleavage of the remaining C/D-rings appear to be highly similar in the aerobic and anaerobic pathways of cholesterol metabolism, and acyl-CoA hydrolase (IpdAB) and acyl-CoA reductase (IpdC) are involved (9, 14).

To date, anaerobic cholesterol catabolism has been less widely investigated than the aerobic 9,10-*seco* pathway and especially the prevalence of the 2,3-*seco* pathway across other bacterial species (2, 4). Wastewater treatment plants have been considered primary engineered ecosystems for removing steroids from environments, mainly through microbial biodegradation in activated sludge (20, 21). Sludge microbial communities have recently drawn large attention due to their high diversity of archaea, bacteria, and fungi (22, 23). So far, only bacteria have been found to display the ability to mineralize the steroid core ring (sterane) under both oxic and anoxic conditions (2, 4, 13). Certain bacteria (24) and fungi (25) are also able to transform cholesterol into androgens through side chain degradation. Thus, cholesterol degradation may be a bioprocess involving cooperation between sludge microorganisms, which would mean that some bacteria and/or fungi degrade the cholesterol side chain and export the recalcitrant sterane for other bacterial degraders to use as carbon sources. Recently, functional traits of microbial communities in wastewater treatment plants have been revealed using metagenomic approaches (26, 27); however, knowledge concerning anaerobic cholesterol degradation in sludge communities remains elusive. Another barrier to further understanding the microbial catabolism of cholesterol is the lack of information on functional aspects at the community level.

Here, we performed a mesocosm experiment by incubating sludges collected from the Dihua Sewage Treatment Plant in Taiwan with exogenous cholesterol under denitrifying conditions. We used ultraperformance liquid chromatography–atmosphere pressure chemical ionization–high-resolution mass spectrometry (UPLC-APCI-HRMS) to identify cholesterol metabolites. Next-generation sequencing techniques, such as 16S amplicon sequencing and metatranscriptome sequencing, were applied to identify cholesterol-degrading bacteria, degradation pathways, and functional genes that respond to recalcitrant cholesterol.

## RESULTS

### Cholesterol and nitrate utilization during mesocosm incubation.

**FIG 2:**
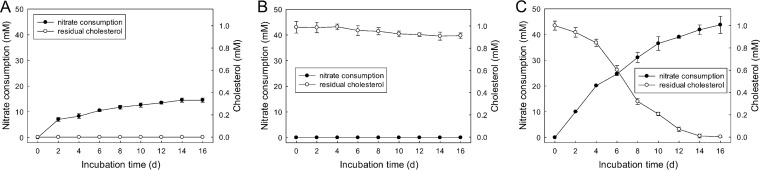
The consumption of cholesterol and nitrate in (A) sludges treated with nitrate only, (B) sludges treated with cholesterol only, and (C) sludges treated with nitrate and cholesterol. Data are shown as means of results from three independent measurements with standard deviations.

In sludges incubated with nitrate and without exogenous cholesterol (SN mesocosm), up to 14.6 ± 0.6 mM nitrate had been slowly consumed after 16 days of incubation (Fig. 2A). No apparent depletion of cholesterol was observed in the anoxic sludges incubated without exogenous nitrate (SC mesocosm; Fig. 2B). In contrast, 40% of the exogenous cholesterol was exhausted within 6 days of incubation under denitrifying conditions (cholesterol and nitrate [SCN] treatment mesocosm), and complete depletion of cholesterol was observed after 14 days and was associated with 43.8 ± 2.4 mM nitrate consumption (Fig. 2C).

### Identification of cholesterol metabolites in the denitrifying sludge.

**FIG 3:**
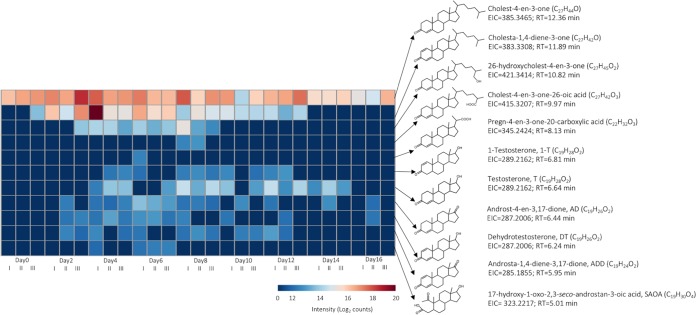
UPLC-APCI-MS analysis of steroid metabolites extracted from sludges treated with cholesterol and nitrate. The log-transformed intensity of each identified metabolite was determined for three independent replicates at each incubation time. The original mass spectrum of each metabolite is listed in Fig. S1.

UPLC-APCI-HRMS analysis of the ethyl acetate extracts revealed that cholesterol was biotransformed from neutral to acidic steroid metabolites in the SCN mesocosm (Fig. 3). The anaerobic transformation of cholesterol to neutral C_27_ metabolites, including cholest-4-en-3-one, cholesta-1,4-diene-3-one, and 26-hydroxycholest-4-en-3-one, occurred within 2 days. Two carboxylic acids, namely, cholest-4-en-3-one-26-oic acid (C_27_) and pregn-4-en-3-one-20-carboxylic acid (C_22_), were also identified. Moreover, various androgen metabolites, including testosterone (T), 1-testosterone (1-T), dehydrotestosterone (DT), androst-4-en-3,17-dione (AD), and androsta-1,4-diene-3,17-dione (ADD), were detected after 2 days of anaerobic incubation with cholesterol and nitrate. A ring-cleavage product, 17-hydroxy-1-oxo-2,3-*seco*-androstan-3-oic acid (SAOA), was also identified. The UPLC retention time and the HRMS spectra of the cholesterol metabolites (see Fig. S1 in the supplemental material) were comparable to authentic standards; thus, we excluded the possibility of detecting structural isomers, which might be observed in metabolite profile analyses based on mass spectrometry.

10.1128/mSystems.00113-18.1 FIG S1 HRMS spectra of cholesterol metabolites extracted from sludges treated with cholesterol and nitrate. Assignment of ion mass spectra was accomplished using authentic standards (deviations of mass pseudomolecular ions [M + H] + <20 ppm). Download FIG S1, TIF file, 1.3 MB. Copyright © 2018 Wei et al. 2018 Wei et al. This content is distributed under the terms of the Creative Commons Attribution 4.0 International license.

### Similarity and diversity of bacterial communities in different sludge treatments.

**FIG 4:**
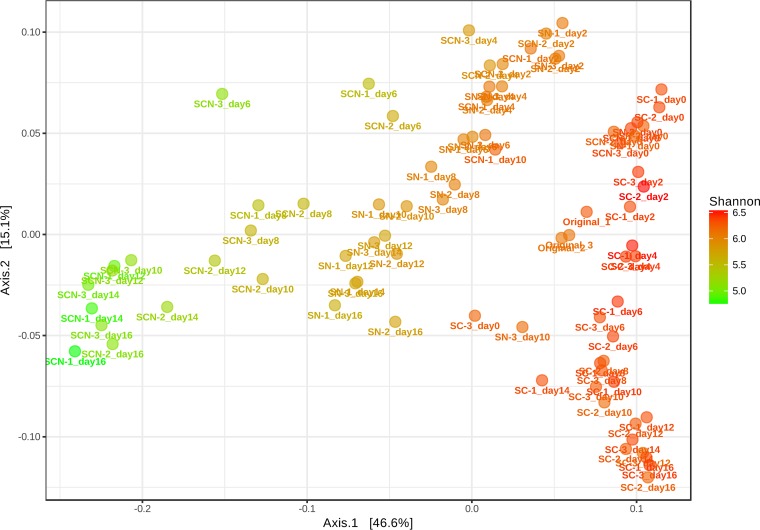
Principal-coordinate analysis (PCoA) for determination of similarities between bacterial communities based on UniFrac distance matrix data (OTU level) from the original sludges and from sludges treated with cholesterol and nitrate (SCN), nitrate only (SN), and cholesterol only (SC) at each incubation time (day 0 to day 16). Colors represent the Shannon diversity index of each data point.

Bacterial diversity was defined using the V3-V4 region of 16S rRNA amplicons on a MiSeq platform (Illumina). Axis 1 (46.6% of total explained variance) of the principal-coordinate analysis (PCoA) largely distinguished among sludge communities treated with cholesterol and nitrate (SCN), cholesterol only (SC), and nitrate only (SN), while axis 2 (15.1% of total explained variance) separated the incubation stages (from day 0 to day 16) of all of the individual treated communities (Fig. 4). Original bacterial communities (Ori) clustered together on the right-hand side of the ordination and were slightly distant from all treated sludge after incubation for 1 h (day 0). All SC communities, which formed highly distinct 16S rRNA gene-defined assemblages after 48 h (day 2) of incubation, clustered together at the right of the coordinate. The SCN and SN communities with incubation stages of shorter duration (day 2 to day 4) were at the top right of the coordinate. However, SN communities with incubation stages of longer duration (from day 6 to day 16) were grouped at the center of the coordinate, whereas the SCN communities with incubation stages of longer duration were at the left of the coordinate. Permutational multivariate analysis of variance (PERMANOVA) (global *R*^2^= 0.36307, *P* value = <0.001) revealed that the weighted UniFrac distance of overall OTUs in each community was significant. Shannon diversity index (*H*’) data indicated that the Ori communities and SC communities displayed the highest community diversity during the whole incubation stage. Notably, the diversity of SCN communities decreased gradually along with incubation time (Fig. 4).

### Temporal changes in bacterial community structure across different treated sludges.

In the Ori samples, *Sphingobacteriia* (21.0%) and *Betaproteobacteria* (18.9%) were the most abundant bacterial species, whereas other bacterial classes comprised only <10% of bacterial communities (see Data Set S1 in the supplemental material). Similarly, *Sphingobacteriia* and *Betaproteobacteria* dominated all treated sludges; however, the relative abundance of *Betaproteobacteria* in SCN communities increased from 21.7% at day 0 to 34.3% at day 16 (Data Set S1). Other bacteria, such as C10-SB1A, SJA-68 *Anaerolineae*, *Acidimicrobiia*, *Alphaproteobactria*, and *Sphingobacteriia*, were slightly enriched, but their abundances decreased in SCN communities by day 16. In SN communities, the relative abundance of *Betaproteobacteria* was 24.9% at day 0; however, no obvious increase was observed during the mesocosm incubation (Data Set S1). SC communities displayed different structural patterns during the incubation. The abundances of *Spirochaetes*, *Anaerolineae*, ValdinHA17, and OPB35 were slightly enriched, and the abundance of OTUs that were manually assigned to the category “others” increased from 9.4% at day 0 to 16.3% at day 16 (Data Set S1).

10.1128/mSystems.00113-18.9 DATA SET S1 The OTU table of bacterial 16S amplicons amplified from treated sludge samples. Download Data Set S1, XLSX file, 2.8 MB. Copyright © 2018 Wei et al. 2018 Wei et al. This content is distributed under the terms of the Creative Commons Attribution 4.0 International license.

**FIG 5:**
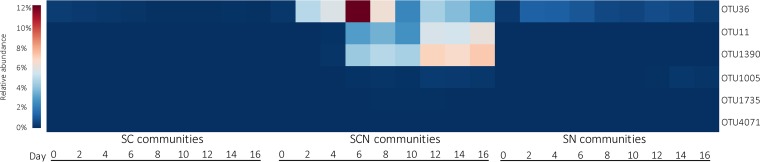
Temporal changes in OTUs classified as genus *Sterolibacterium* from sludges treated with cholesterol only (SC), cholesterol and nitrate (SCN), and nitrate only (SN) during mesocosm incubation (day 0 to day 16). Data are shown as averages of triplicates.

We observed that among *Betaproteobacteria*, relative abundances of members of genus *Sterolibacterium* (OTU11, OTU36, and OTU1390) were highly enriched during the incubation in SCN communities, increasing from 0.2% at day 0 to 16.8% at day 16 (Fig. 5; see also Fig. S2). The relative abundance of OTU36 was greatly enriched in the period since day 2 (to 12.3% at day 6), whereas the relative abundances of OTU11 and OTU1390 were increased only after day 6. Notably, the relative abundance of OTU36 decreased to 6.4% at day 8, and OTU11 and OTU1390 became more abundant (6.2% and 7.3%, respectively) than OTU36 (3.0%) in SCN communities at day 16 (Fig. 5). OTU1005, OTU1735, and OTU4071 were also classified as *Sterolibacterium*, but no enrichment was observed in these OTUs (Fig. 5). In SN communities, the abundance of *Sterolibacterium* increased from only 0.3% at day 0 to 1.2% at day 4 (Fig. S2), primarily due to OTU36, while the abundance of *Sterolibacterium* in SC communities remained extremely low (<0.1%) (Fig. 5; see also Fig. S2). The normalized counts of each OTU in different mesocosm treatments are listed in Data Set S1.

10.1128/mSystems.00113-18.2 FIG S2 The heat map represents the relative abundance of each betaproteobacterial genus from sludges treated with cholesterol only (SC), cholesterol and nitrate (SCN), and nitrate only (SN) at each incubation time point. “Ca” denotes “*Candidatus*,” and “U” denotes “unclassified.” Download FIG S2, TIF file, 1 MB. Copyright © 2018 Wei et al. 2018 Wei et al. This content is distributed under the terms of the Creative Commons Attribution 4.0 International license.

### General functional characterization of the sludge communities.

The total protein-encoding transcripts derived from SCN1_day10, SCN3_day10, SN1_day10, and SN3_day10 were functionally annotated against the KEGG database, and up to 129,720 sequences were assigned to 4,898 different KEGG orthologs (KOs). The overall functional profiles were different between two communities (Fig. S3), and the functional diversity—Shannon *H*′ (based on the counts per million [CPM] determined for each KO)—in SCN communities (7.00 ± 0.04) was higher than in SN communities (6.67 ± 0.26). Also, the mean values of CPM for each KEGG pathway in SCN communities were more abundant than in SN communities, except for pathways for signaling molecules and interactions and for viruses. Of particular interest were the transcripts mapping to pathways for cell motility, transport, and biosynthesis of vitamins and cofactors, and the overall expression levels of these pathways were higher in SCN communities than in SN communities (Fig. S3).

10.1128/mSystems.00113-18.3 FIG S3 Functional profiles in SCN and SN communities. (A) The abundance (log cpm) of each of the KEGG orthologs (KOs) (4,898 in total). Cluster analysis revealed that the levels of gene expression in two communities were different. Average linkage clustering was conducted using Euclidean distance. (B) The abundance (in counts per million [CPM]) of pathways involved in transporters, cellular motility, and vitamin and cofactor biosynthesis. Download FIG S3, TIF file, 2.3 MB. Copyright © 2018 Wei et al. 2018 Wei et al. This content is distributed under the terms of the Creative Commons Attribution 4.0 International license.

**FIG 6:**
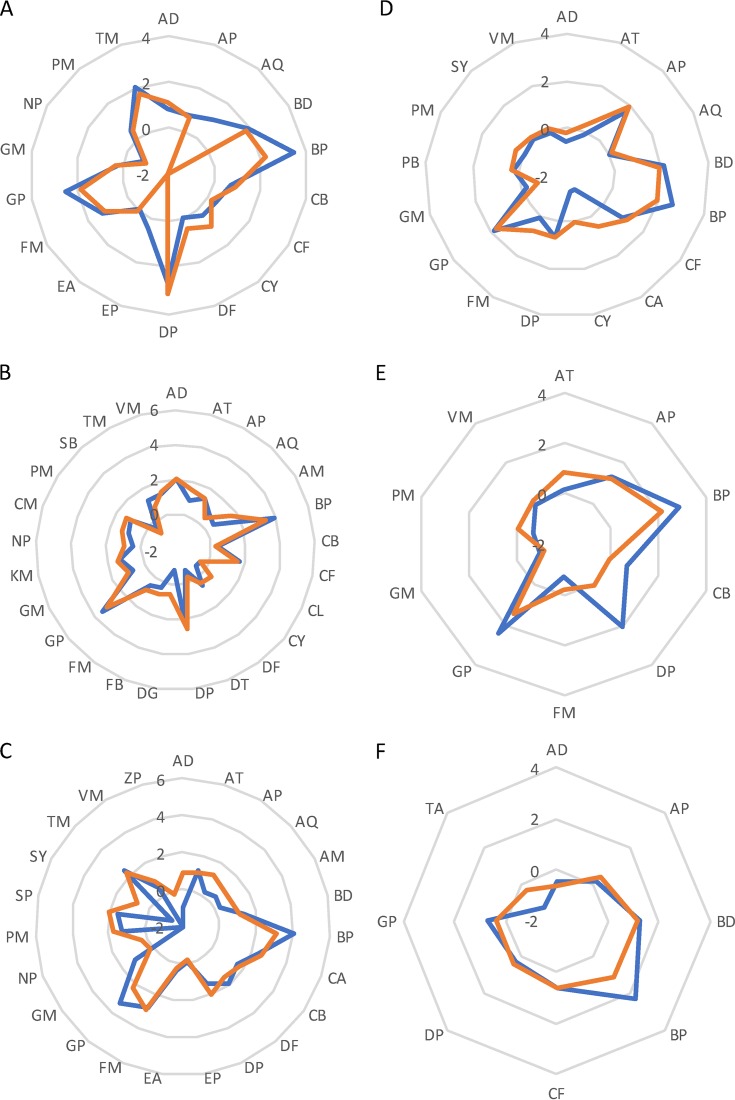
Taxonomic contribution of each KEGG pathway involved in (A) chemotaxis, (B) type IV pili, (C) flagellar, (D) uroporphyringonen and ALA synthesis, (E) cobalamin *de novo* synthesis, and (F) cobalamin salvage synthesis in SCN (blue) and SN (orange) communities. Each arm of each spider chart indicates the mean of log-transformed counts per million (CPM) for a given bacterial phylum. The −2 value represents a mean CPM value of 0 before log transformation. AQ, *Aquificae*; BP, *Betaproteobacteria*; DP, Deltaproteobacteria; GP, *Gammaproteobacteria*; FM, *Firmicutes*; TM, *Thermotogae*. Other phyla denoted by two-letter codes are listed in Table S4.

The KOs related to cell motility were categorized into chemotaxis, type IV pilus assembly and twitching, and flagellar assembly. We observed that, in SN communities, genes involved in chemotaxis sensing were mainly expressed by *Betaproteobacteria*, *Deltaproteobacteria*, *Gammaproteobacteria*, and *Thermotogae*; however, under conditions of cholesterol treatment, *Betaproteobacteria*, *Gammaproteobacteria*, *Thermotogae*, and *Aquificae* highly expressed chemotaxis genes (Fig. 6A). Similarly, in SN communities, the abundances of genes for type IV pili and flagella were primarily contributed to by *Proteobacteria* (except *Alphaproteobacteria*), *Firmicutes*, and *Thermotogae*, whereas beta- and gammaproteobacterial genes were most highly expressed in sludges treated with cholesterol (Fig. 6B and C). The transcripts encoding transporters for nitrate/nitrite (K02575), long-chain fatty acid (K06076), molybdate (K02017, K02018, and K02020) and the TonB-ExbB-ExbD complex transporter (K03559, K03561, and K03832) were dominated by *Betaproteobacteria* in SCN communities, although in SN communities the major contributors to each transporter synthesis were disparate (Fig. S4A to D). Notably, *Gammaproteobacteria* mainly accounted for expression of cobalt transporters (K02006 to K02009) in SCN communities, but enrichment of betaproteobacterial genes was greater (Fig. S4E).

10.1128/mSystems.00113-18.4 FIG S4 Taxonomic contribution of KEGG pathways, including (A) nitrate/nitrite transporter, (B) long-chain fatty acid transporter, (C) molybdate transporter, (D) TonB transporter, (E) cobalt transporter, (F) pantothenate and CoA, (G) thiamine, (H) nicotinate and nicotinamide, (I) vitamin B6, (J) riboflavin, (K) ubiquinone and other terpenoid-quinones, (L) biotin, (M) retinol, (N) folate, and (O) lipoic acid pathways, in SCN (blue) and SN (orange) communities. Each arm in each chart indicates the mean of log-transformed counts per million (CPM) of a given bacterial phylum. The −2 value represents value of mean CPM of 0 before log transformation. Phyla denoted by two-letter codes are listed in Table S4. Download FIG S4, TIF file, 1.4 MB. Copyright © 2018 Wei et al. 2018 Wei et al. This content is distributed under the terms of the Creative Commons Attribution 4.0 International license.

We also targeted taxonomic contributions for biosynthesis of vitamin and cofactors between SN and SCN communities based on KEGG data. *Proteobacteria* and *Bacteroidetes* generally accounted for synthesis of nicotinate and nicotinamide, pantothenate and CoA, retinol, thiamine, ubiquinone, riboflavin, biotin, vitamin B_6_, lipoic acid, and folate (molybdopterin cofactor), with minor contributions from *Aquificae*, *Chloroflexi*, *Chlorobi*, and *Thermotogae* (Fig. S4F to O). Under conditions of treatment with cholesterol, *Betaproteobacteria* highly expressed genes involved in synthesizing the vitamins and cofactors mentioned above (Fig. S4F to O); however, striking differences were observed in the expression patterns of cobalamin biosynthesis genes. Prior to cobalamin *de novo* biosynthesis, precursors (including δ-aminolevulinate [ALA] and uroporphyrinogen III) are required. *Betaproteobacteria* and *Gammaproteobacteria* highly expressed genes involved in precursor synthesis in SCN communities (Fig. 6D), and genes related to cobalamin *de novo* biosynthesis were highly expressed by *Betaproteobacteria*, *Deltaproteobacteria*, and *Gammaproteobacteria* (Fig. 6E). On the other hand, *Proteobacteria* mainly accounted for the cobalamin salvage pathway (K16092, K02231, and K19221), and betaproteobacterial genes were highly expressed in SCN communities (Fig. 6F). Archaeal signatures were also recovered in certain vitamin and cofactor synthesis pathways, but they comprised a very small fraction of overall expression levels.

### Identification of the catabolic genes for anaerobic cholesterol degradation.

**FIG 7:**
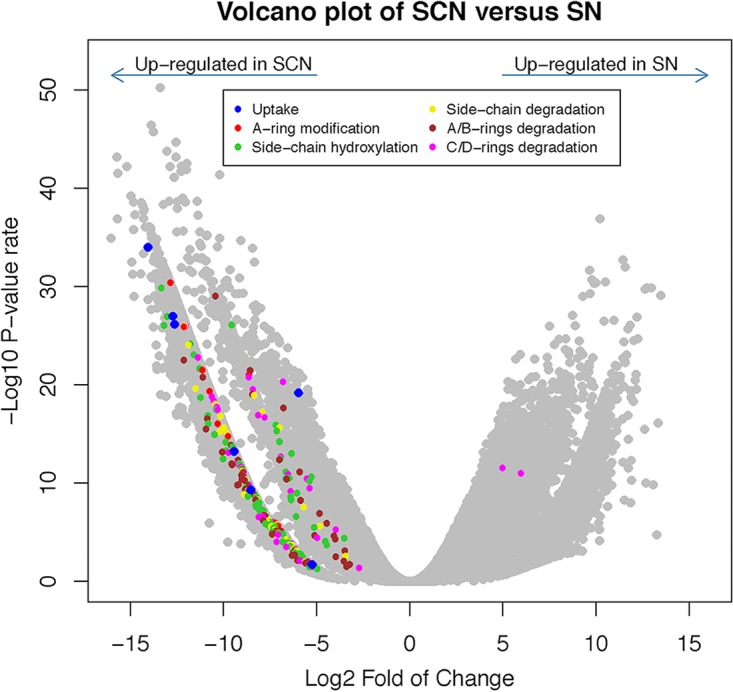
Volcano plot of significantly expressed transcripts involved in anaerobic cholesterol degradation between SCN and SN samples. Up to 257 transcripts involved in cholesterol uptake, A-ring modification, C25 hydroxylation, side chain degradation, and sterane degradation were identified, and only two transcripts annotated as C/D-ring degrading were expressed in SN samples.

On the basis of the RefSeq non-redundant protein database, up to 222,329 genes were functionally annotated in our metatranscriptome data. According to differential expression analysis results, only 30,382 genes were significantly expressed in SCN communities, whereas in SN communities 27,959 transcripts were significantly expressed. Among these transcripts, we observed that 257 transcripts significantly expressed in SCN communities were putatively involved in anaerobic cholesterol degradation and that two were significantly expressed in SN communities (Fig. 7). Information on transcripts, including fold change, adjusted *P* value, and annotation source (RefSeq accession), is listed in Data Set S2.

10.1128/mSystems.00113-18.10 DATA SET S2 The transcripts involved in the 2,3-*seco* pathway for cholesterol degradation identified in SCN and SN sludge samples. Download Data Set S2, XLSX file, 0.1 MB. Copyright © 2018 Wei et al. 2018 Wei et al. This content is distributed under the terms of the Creative Commons Attribution 4.0 International license.

Seven transcripts encoding the FadL proteins were significantly expressed in SCN, which might be involved in cholesterol uptake. Ten transcripts were annotated as bifunctional beta-hydroxysteroid dehydrogenase/isomerase AcmA. Additionally, up to 81 transcripts were identified as molybdopterin-containing subunits of C25DH (S25dA1 to S25dA7 [S25dA1–7]), as well as its heme B-containing subunit (S25dC4) and a putative chaperone (S25dD4) responsible for the anaerobic hydroxylation at C-25 of the sterol substrates. Side chain degradation of 26-hydroxycholest-4-en-3-one to downstream metabolite AD requires three cycles of β-oxidation reactions, and the enzymes aldehyde dehydrogenase (ALDH), acyl-CoA synthetase (ACS), acyl-CoA dehydrogenase (ACAD), enoyl-CoA hydratase (ECH), and aldolase (ALD) are essential for each cycle. Several transcripts, annotated as ACAD, involved in C_26_ intermediate degradation; ACS and ACAD, involved in C_24_ intermediate degradation; and ALDH, ACS, ACAD, ECH and ALD, involved in C_22_ intermediate degradation, were significantly expressed.

We annotated 6 and 16 transcripts as 3-ketosteroid-Δ^1^-dehydrogenase (AcmB) and 3-ketosteroid-Δ^4^-reductase, respectively. We also observed differential expression levels in homologues of the *atcABC* and acyl-CoA synthetase genes specifically responsible for the activation of androgen metabolites and 3aα-*H*-4α(3'-propanoate)-7aβ-methylhexahydro-1,5-indanedione (HIP) in SCN, respectively. The microbial degradation of C/D-rings requires the β-oxidation reactions and hydrolytic cleavage. We identified significantly expressed transcripts involved in these bioprocesses in the SCN community, including ACAD, ECH, and thiolase (TL) as well as IpdAB and IpdC. The total level of expression abundance of the functional genes mentioned above was also calculated using aggregated CPM of each homologue. The homologues of FadL and S25dA1 were the most abundant, whereas the level of expression of S25dC4 homologues was the lowest (see Table S1 in the supplemental material).

10.1128/mSystems.00113-18.5 TABLE S1 The expression level (count per million [CPM]) of each protein homologue involved in anaerobic cholesterol degradation in SCN communities. The mean value was calculated from the sum of CPM values for each transcript annotated as a given enzyme in two biological replicates of SCN1 and SCN3. Download Table S1, DOCX file, 0.01 MB. Copyright © 2018 Wei et al. 2018 Wei et al. This content is distributed under the terms of the Creative Commons Attribution 4.0 International license.

### Phylogenetic analysis of fragments of 16S rRNA genes, *atcA*, and *s25dA1–8*.

**FIG 8:**
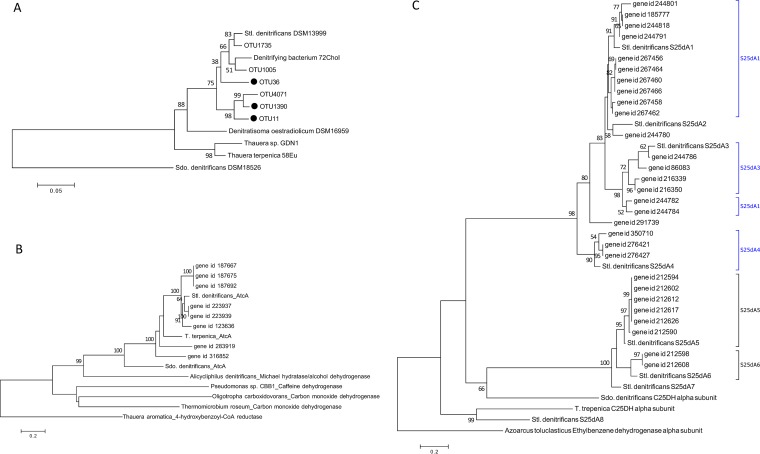
(A) Maximum likelihood tree of 16S rRNA gene of steroid-degrading anaerobes and *Sterolibacterium*-like OTUs. Only enriched OTUs are highlighted. (B) AtcA and its homologues and other molybdopterin-containing xanthine oxidase family members. (C) Alpha subunit of C25DH and its homologues. Subunits (A1 to A4) displaying specificity for side chain substrates are highlighted in blue. Branch support of higher than 50% of bootstrapping time is shown. Details for all reference sequences are provided in Table S3.

The maximum likelihood tree showed that the gammaproteobacterium Steroidobacter denitrificans DSM18526 (Sdo. denitrificans) formed a lineage separate from those of other steroid-degrading betaproteobacteria and *Sterolibacterium*-like OTUs (Fig. 8A). OTU1735 and OTU1005 displayed the highest 16S rRNA sequence similarity to Stl. denitrificans and strain 72Chol (28)—with 98.1% and 97.0% identity, respectively (Table 1)—and formed a clade distinct from OTU11, OTU36, OTU1390, and OTU4071 (Fig. 8A), which displayed low 16S rRNA sequence similarity to DSM 13999 and 72Chol (92.2% to 95.1%) (Table 1). All the selected transcripts annotated as AtcA homologues formed a distinct clade with AtcA derived from steroid-degrading anaerobes, whereas other molybdopterin-containing proteins presenting different degradation functions were grouped into other lineages (Fig. 8B). In the maximum likelihood tree of the C25DH alpha subunit, two major clades—S25dA1–4 and their homologues and S25dA5–7 and their homologues—were separated. Also, each subunit and its homologues were grouped into same clade, except S25dA2 and its homologue. Notably, C25DH of Sdo. denitrificans forms an individual lineage and subunit of S25dA8 of Stl. denitrificans and C25DH of Thauera terpenica 58Eu was clustered in the same clade (Fig. 8C).

**TABLE 1:** Pairwise analysis of 16S rRNA gene fragments derived from cholesterol-degrading *Stl. denitrificans* DSM 13999 and isolate 72Chol and of *Sterolibacterium-*like OTUs

Isolate or OTU	% 16S						
	Chol 72	DSM 13999	OTU11	OTU36	OTU1005	OTU1390	OTU1735
DSM 13999	96.4						
OTU11	93.4	94.1					
OTU36	95.1	95.3	93.7				
OTU1005	97.0	96.7	93.7	96.0			
OTU1390	92.3	93.9	95.8	92.5	93.0		
OTU1735	96.3	98.1	94.8	95.3	97.0	93.4	
OTU4071	92.3	93.0	95.8	92.5	93.0	96.7	93.4

## DISCUSSION

Compared to aerobic sterol metabolism, data from investigations performed in anoxic environments are relatively scant. Taylor et al. (29) reported for the first time that bacteria are capable of degrading cholesterol in sediments under denitrifying and sulfate-reducing conditions. Although certain fermentative bacteria were able to reduce cholesterol into coprostanol (30, 31), we did not observe the production of coprostanol or high levels of enrichment of fermentative bacterial OTUs in the cholesterol-amended sludge without nitrate. Thus, we excluded the possibility of a role of fermentative bacteria in cholesterol catabolism in analyses of our anoxic sludge. On the other hand, cholesterol exhaustion was accompanied by the consumption of nitrate, indicating that denitrifying bacteria degraded cholesterol in the anoxic sludge.

To date, the 2,3-*seco* pathway has been the only anaerobic cholesterol degradation pathway identified, and catabolic genes and the corresponding metabolites have been identified in denitrifying Stl. denitrificans (13–19). The metabolite and putative degradation genes identified in this study completely mirrored the cholesterol transformation steps involved in sterane modification (15, 17) and A ring degradation (14, 19), although some metabolites or genes related to C25 hydroxylation, side chain degradation, and C/D-ring degradation were missing. The transformation of 25-hydroxycholest-4-en-3-one to 26-hydroxycholest-4-en-3-one appeared to be the most highly rate-limiting step in the overall pathway, accumulating 25-hydroxycholest-4-en-3-one in Stl. denitrificans cultures (18). We did not detect this particular 25-hydroxysteroid intermediate in SCN samples; instead, homologues of the C25DH molybdopterin-containing subunits (S25dA1–A7) were identified, suggesting the occurrence of anaerobic 25-hydroxylation in the SCN samples. The hydrophilic CoA-ester intermediates were absent during side chain degradation, and this was partially due to the ethyl acetate extraction procedure and poor ionization of these hydrophilic metabolites in the APCI mode used in this study (32). Instead, we identified C_26_ and C_22_ carboxylic intermediates and ACS responsible for the activation of C_24_ and C_22_, indicating the degradation of cholesterol side chain through β-oxidation reactions in anoxic sludge communities. Genes putatively involved in the degradation of C/D-rings were also identified in SCN samples, supporting the theory that the processes of hydrolytic cleavage of C/D-rings appeared to be similar in aerobic and anaerobic steroid-degrading bacteria (9, 14). Overall, our data indicated that bacterial communities in this denitrifying sludge adopt the 2,3-*seco* pathway to degrade cholesterol and that the sequence of this processes—degradation of cholesterol side chain prior to sterane degradation from the A-ring—is highly similar to those occurring in Stl. denitrificans (12, 13).

Sludge in wastewater treatment plants comprised complex sterols, among which fecal sterols and cholesterol are generally the most abundant (21, 33). The content of exogenous cholesterol in our mesocosms was approximately 2 orders of magnitude higher than that measured in our original sludge samples (4.26 ± 0.29 mg/liter). We observed high levels of enrichment of OTU11, OTU36, and OTU1390 (all classified as *Sterolibacterium* and *Betaproteobacteria*) since day 6 in the SCN samples, but the levels of these OTUs were not enriched in other mesocosms. A gene in a denitrifying betaproteobacterium, strain 72Chol, the first isolate found to be capable of degrading cholesterol anaerobically (28), displayed high (96.5%) similarity to the 16S rRNA gene in Stl. denitrificans (10). The phylogenetic analyses showed that OTU11, OTU36, and OTU1390—enriched by cholesterol—might be novel denitrifying bacteria with the capacity to degrade cholesterol in anoxic sludge. Surprisingly, OTU1005 and OTU1735—with higher 16S rRNA sequence similarity to 72Chol and Stl. denitrificans—were not enriched in SCN samples, indicating that cholesterol might not be the preferable growth substrate for OTU1005 and OTU1735 in this given mesocosm environment.

Both Stl. denitrificans and 72Chol strains exhibited extremely narrow spectra of substrate utilization. Stl. denitrificans was able to grow only with various fatty acids, sterols (10, 14), and androgens (11, 34). However, instead of *Sterolibacterium* spp., *Thauera* spp. were identified as the major androgen degraders in anoxic sludge derived from the same treatment plant (19). In this study, we detected androgen metabolites in the SCN samples, but enrichment of *Thauera* spp. and androgen-degrading *Steroidobacter* spp. did not occur (35). These findings suggested that cholesterol degradation in the anoxic sludge may not be a cooperative catabolic process performed by side chain degraders and sterane degraders.

The ability to degrade sterol side chains might be associated with the substrate specificity of C25DH. The hydroxylation of C_25_ is an essential bioprocess prior to β-oxidation of the sterol side chain (16). In fact, Stl. denitrificans harbors eight alpha subunits (molybdopterin-containing subunits) of C25DH, and the substrate specificity of each subunit (S25dA1–8) has been studied through proteome analysis (14). We identified homologues of S25dA1–A7, but the expression level of S25dA1 homologues in our cholesterol-treated sludge was highest; this is congruent with previous studies that indicated that S25dA1 is responsible for the side chain hydroxylation of cholest-4-en-3-one due to its high substrate specificity (14, 36). Interestingly, our maximum likelihood tree clearly separated subunits with side chain specificity (S25dA1–4) and subunits without specificity (S25dA5–A7) into two lineages and grouped homologues of each subunit into distinct clades (except S25dA2). This suggests that each C25DH alpha subunit is highly conserved. Although Sdo. denitrificans and T. terpenica contain C25DH genes, their roles in anaerobic steroid degradation remain unclear (19). Our phylogenetic analysis shows that these two enzymes are similar to S25dA8 of Stl. denitrificans, whose function is not relevant to this particular hydroxylation bioprocess of the sterol side chain (14). The incapability of using cholesterol as a growth substrate in Sdo. denitrificans; T. terpenica (19, 35); and OTU1005, OTU1735, and OTU4071 might result partially from the lack of S25dA1. The *atcA* gene was proposed to serve as a biomarker to interrogate anaerobic steroid degradation due to its high dissimilarity to other xanthine oxidase enzyme families as well as its high substrate specificity (19). Accordingly, we propose that *s25dA1* can be applied as a biomarker to detect cholesterol side chain degradation in environmental samples. For example, the GeoChip microarray that targets microbial metabolism has been extensively applied to monitor microbial activities (37).

Chemotaxis is a major mechanism for sensing environmental signals, promoting movement of bacterial cells toward beneficial substrates or away from harmful substances (38). The addition of cholesterol appears to induce chemotaxis genes in *Betaproteobacteria*, *Gammaproteobacteria*, and *Thermotogae*, resulting in cell motility for this hydrophobic growth substrate (38, 39). A similar observation was made for strain 72Chol, which displayed motility after being grown in cholesterol (34). The chemosensory machinery of steroid-degrading anaerobes has yet to be addressed, but some studies have demonstrated that chemotaxis can increase the bioavailability of substrates and enhances the efficiency of biodegradation (40). Knowledge of the mechanism of uptake of Gram-negative anaerobes for hydrophobic cholesterol remains elusive. The FadL is an outer membrane protein responsible for hydrophobic substrate transport across the hydrophilic lipopolysaccharide layer in Gram-negative bacteria via a facilitated diffusion mechanism (41). The high level of induction of betaproteobacterial *fadL* genes in cholesterol-grown Stl. denitrificans (13, 14) and in cholesterol-treated denitrifying sludge suggested that long-chain fatty acid transporters may be involved in cholesterol uptake in our sludge communities.

The chemically defined medium for culturing Stl. denitrificans contains various vitamins (10). These micronutrients are essential for cellular metabolism and play important roles in biodegradation in sludge communities (42). We did not add any vitamin and cofactor solutions into the mesocosms; however, genes involved in micronutrient uptake and the synthesis of vitamins and cofactors were highly expressed in cholesterol-treated sludge, and the target species of annotation output were mostly Stl. denitrificans. In fact, the Stl. denitrificans genome contains genes for vitamin and cofactor biosynthesis, some of which are required for cholesterol anaerobic degradation—e.g., CoA and molybdopterin, which are important for β-oxidation and Mo-containing enzymes (C25DH and AtcABC), respectively. Overall, we inferred that cholesterol-degrading betaproteobacteria in sludge were capable of transporting precursors and synthesizing these vitamin and cofactors independently. The most intriguing aspect is cobalamin biosynthesis in cholesterol-treated sludges because of taxonomic discrepancy in the *de novo* and salvage synthesis pathways. Vitamin B12 (cyanocobalamin) is the most complicated organometallic (cobalt-inserted) cofactor (43) and is crucial for biochemical reactions in many organisms (44). B_12_ biosynthesis, starting from uroporphyrinogen formation, requires up to 30 genes for its completion, and only certain eubacteria and archaea possess the machinery for this particular *de novo* synthesis process. In contrast, a salvage synthesis pathway is adopted for bacteria that do not harbor *de novo* synthesis machinery (43, 45). We cannot exclude the possibility that betaproteobacterial degraders are able to synthesize cobalamin *de novo*. However, the greater expression of betaproteobacterial genes in the salvage synthesis pathway indicates that the cobalamin required by some of cholesterol-degrading bacteria might be provided by cobalamin-synthesizing *Deltaproteobacteria*, *Gammaproteobacteria*, or *Betaproteobacteria*. This fits the scenario that public goods are usually more beneficial to nonproducers, and the quorum sensing system controls the production of public goods in microbial communities (46).

The extremely low abundance of OTU11, OTU36, and OTU1390 (<0.1%) in the original sludge samples indicates that they were likely members of the rare biosphere (47–49). The rarity of these community members might result from low concentrations of the growth substrate in their original environments (the cholesterol content was 4.26 ± 0.29 mg/liter in sludge) because these OTUs were highly enriched by supplementations of cholesterol (approximately 386 mg/liter) in mesocosms. Similarly, several bacterial strains responsible for anaerobic androgen degradation in sludge and estuarine sediments also had very low abundances before the addition of the androgen substrate (19, 50). This is congruent with the notion that members of the rare biosphere become dominant only when they encounter favorable nutrients or conditions (48, 51–53). The ecological roles of anaerobic steroid degraders in natural environments may not be significant due to their low abundance (2); however, we have demonstrated that in communities, anaerobic steroid degraders do not share functional redundancy in degradation of side chains and sterane, supporting the paradigm that members of the rare biosphere serve as genetic reservoirs, exhibiting unique functional traits in biogeochemistry and bioremediation (49). The capability of vitamin and cofactor biosynthesis in anaerobic cholesterol-degrading bacteria in sludge communities is much more unappreciated, especially in cobalamin synthesis. The Stl. denitrificans genome contains only genes for the cobalamin salvage pathway according to BlastKOALA output from KEGG (data not shown), and the role of cobalamin in this microorganism may be related to survival in anoxic environments using class II (cobalamin-dependent) ribonucleotide reductase for DNA synthesis (54). The entire cobalamin synthesis process and its roles in cholesterol-degrading bacteria were not investigated comprehensively in this study, but the significance of cobalamin in shaping the structure and functioning of microbial communities has been confirmed (44, 55).

### Conclusion and outlook.

In these highly diverse sludge communities, only three betaproteobacterial OTUs (∼0.1% of total abundance) that are phylogenetically distant from Stl. denitrificans are denitrifying cholesterol degraders adopting the 2,3-*seco* pathway. Although other bacteria, such as *Gammaproteobacteria* and *Thermotogae*, putatively displayed chemotaxis and cellular motility toward cholesterol in this study, the degradation capacity for cholesterol appears to be associated with uptake protein, FadL, side chain-specific molybdoenzyme C25 dehydrogenase, and several β-oxidation enzymes. Another Mo-containing 1-testosterone hydratase/dehydrogenase that catalyzes the A-ring degradation of androgen metabolites appears to be common in cholesterol- and testosterone-degrading anaerobes. Our metatranscriptome analysis also indicated that transporter and cofactor synthesis genes are accessories to this anaerobic degradation process, providing an insight into the functional diversity of anaerobic cholesterol catabolism in microbial communities from an engineered ecosystem.

Sludge communities are known to contain diverse steroid-degrading anaerobes (10, 19, 34, 35). However, much less is known about their distribution in natural environments. To date, apart from wastewater treatment plants, the signatures of *Sterolibacterium*-like bacteria have been recovered from contaminated groundwater (56), lake sediment (57), cattle manure (58), and the gut of medicinal leeches (59); but their roles in these ecosystems are largely unknown. Anthropogenic inputs may allow microorganisms related to industrial activities to thrive (60). Hence, we may envisage anaerobic steroid-degrading bacteria being prevalent in anoxic environments—such as sediments of freshwater ecosystems, where steroid input is either natural or industrial in origin—and supporting diverse catabolism and transformation mechanisms (50). Due to their low abundance in environments, a combination of enrichment (mesocosm) and metagenomic approaches would be essential to elucidate functional capacities of the rare biosphere in microbial communities.

## MATERIALS AND METHODS

### Sample collection.

The Dihua Sewage Treatment Plant is the largest municipal wastewater treatment plant in the Taipei metropolitan area (approximately 3 million residents), and receives wastewater from manufacturing industries, medical industries, animal husbandry, and influent of groundwater (500,000 m^3^ day^−1^). This plant is designed to employ a combination of oxic and anoxic bioprocesses for toxic carbon and nitrogen removal. The specification of the Dihua Plant and the efficiency of pollutant removal were described previously (20, 61). Sludge (∼5 liters) was collected on 22 March 2017 from a single anoxic tank (a denitrifying tank; the dissolved oxygen level is 0.2 to 0.5 mg liter^−1^) and was stored in sterilized narrow-neck serum bottles to avoid exposure to oxygen. The sludge was collected as triplicates and was carried to the laboratory within 1 h at 4°C for mesocosm experiments performed immediately. Additional sludge (∼50 ml) was also collected as triplicates and snap-frozen by treatment with liquid nitrogen to retain the original nucleic acid status for extraction (62).

### Mesocosm incubation of anoxic sludge with cholesterol and nitrate.

Anoxic sludge (∼1 liter) was transferred into 1-liter sterilized serum bottles in laminar flow and was incubated with cholesterol (1 mM) and sodium nitrate (initially 10 mM) (abbreviated “SCN”). Sludge was also incubated with cholesterol (1 mM) only (SC) and with sodium nitrate (10 mM) only (SN) as controls. These mesocosms were prepared under an anoxic condition by purging nitrogen gas (80% [vol/vol]) and carbon dioxide (20% [vol/vol]) into bottles sealed by butyl rubber stoppers. Ascorbate (a reducing agent) and resazurin (a redox indicator) were not added because these compounds could be used as carbon and energy sources for sludge bacteria (19). Each treatment was performed in triplicate, and the reaction mixtures were incubated at room temperature with agitation until cholesterol was completely degraded. Sludge samples (10 ml) were withdrawn from each mesocosm every 2 days using syringes for total DNA/RNA extraction and metabolite analyses. The consumption of nitrate was monitored using Spectroquant nitrate test kit HC707906 (Merck, Germany). Sodium nitrate (10 mM) was resupplied when the nitrate was depleted until the cholesterol was completely degraded. The values of total nitrate consumption are presented as means with standard deviations calculated from triplicate data.

### Determination of free sterols.

Free sterol content in the sludge samples was determined using the *o*-phthaladehyde method (63) with slight modifications. The sludge samples (100 ml) were extracted by the use of ethyl acetate three times (50). The total extracts were then dried using a centrifugal evaporator and were redissolved in 2 ml of glacial acetic acid containing 1 mg of *o*-phthaldialdehyde. After incubation was performed at room temperature for 10 mins, 1 ml of concentrated sulfuric acid was added and mixed immediately. After further incubation at room temperature for 10 min, the absorbance was measured photometrically at 590 nm. The sterol content levels are presented as means with standard deviations calculated from triplicate data.

### UPLC–APCI–HRMS analysis of steroid metabolites.

Metabolites from treated sludge (including SCN, SC, and SN samples) were extracted using ethyl acetate as previously described (50). The crude extracts were then applied to the UPLC-MS with UPLC coupled to an APCI-mass spectrometer to identify and quantify cholesterol metabolites. Mass spectrum data were collected in positive APCI mode for separate runs on an Orbitrap Elite Hybrid Ion Trap-Orbitrap mass spectrometer (Thermo Fisher Scientific, Waltham, MA, USA) operated in scanning mode (50 to 500 *m/z*). Separation was achieved on a reversed-phase C_18_ column (Acquity UPLC BEH C18; Waters) (1.7-μm pore size, 100 by 2.1 mm). The separation conditions were applied as previously described (13). The predicted elemental composition of individual steroid metabolites was calculated using Xcalibur mass spectrometry software (Thermo Fisher Scientific). The MS counts of each metabolite were visualized as a heat map using the Web-based tool MetaboAnalyst (64). The following authentic standards were purchased from Sigma-Aldrich for the UPLC analysis: cholesterol, T, DT, AD, and ADD. Cholest-4-en-3-one, cholesta-1,4-diene-3-one, and pregn-4-en-3-one-20-carboxylic acid were purchased from Steraloids (Newport, RI, USA). Cholesta-4-en-3-one-26-oic acid and 26-hydroxycholest-4-en-3-one were purchased from Avanti Polar Lipids (Alabaster, AL, USA). 1-Testosterone and 17-hydroxy-1-oxo-2,3-*seco*-androstan-3-oic acid (SAOA) were obtained from a previous study (13).

### DNA extraction and 16S rRNA amplicon sequencing.

Bacterial communities from the original anoxic tank (“Ori”; *n* = 3) and community structures with temporal changes (from day 0 to day 16; nine time points) under three different treatment conditions (SCN, *n* = 27; SC, *n* = 27; SN, *n* = 27) were identified using an Illumina MiSeq platform. DNA was extracted from the sludge using a PowerSoil DNA isolation kit (Qiagen, Germany). The 16S amplicon libraries targeting the V3-V4 regions of 16S rRNA genes were prepared according to the Illumina 16S Metagenomic Sequencing Library Preparation Guide with a gel purification approach for expected PCR products (approximately 445 bp) using a Tools EasyPrep gel and PCR extraction kit (Tools, Taiwan) as previously described (19). In total, 84 libraries were generated and their profiles were analyzed by the use of a BioAnalyzer 2100 with High Sensitivity DNA kit (Agilent, USA). To ensure the evenness of pooling, all 84 libraries were subjected to quantitative PCR (qPCR) for normalization using a Kapa library quantification kit to obtain molar concentrations. For sequencing, the pooled library was run on an Illumina MiSeq V2 sequencer with MiSeq reagent kit V3 (paired end [PE]; 2 by 300 bp) at the High-Throughput Genomics Core Laboratory at the Biodiversity Research Center, Academia Sinica.

### Preprocessing and taxonomic assignment for 16S sequences.

MiSeq sequencing generated 29,310,906 total reads from 84 sludge samples. One of the 16S amplicons, SN-2_day14, generated only 3,198 reads with a lower mean quality score of 20.27 and was excluded from data processing and analyses. USEARCH (65) and mothur (66) were applied to process raw 16S rRNA sequences as previously described (67). The representative sequences of the OTUs, 6,109 in total, were taxonomically assigned against SILVA 128 (68) using mothur v1.35.1. The relative abundances determined for each bacterial class, genus, or OTU were based on average abundance data derived from triplicate data except SN_day14. Abundance levels of less than 1% at the class level were manually assigned as representing “others.”

### Statistical analyses for community structure.

The number of representative OTUs in each sample ranged from 64,255 to 251,724. The Web-based program MicrobiomeAnalyst (in the Marker Data Profiling module) was applied for downstream analyses (69). The OTU counts from all samples were first normalized via cumulative sum scaling (70). Similarities between microbial communities from differently treated sludge communities (Ori, SCN, SN, and SC) were determined using principal-coordinate analysis (PCoA) based on the weighted UniFrac distance matrix (OTU level). Differences between communities in weighted UniFrac distances were tested using permutational multivariate analysis of variance (PERMANOVA). Alpha diversity and Shannon diversity index (*H*′) data were also calculated for all communities individually.

### Total RNA extraction and metatranscriptome sequencing.

To compare the expression level differences among functional genes involved in cholesterol catabolism under denitrifying conditions, total RNA was extracted from the SCN_day10 and SN_day10 samples using an RNeasy PowerSoil total RNA kit (Qiagen, Germany). The quality of total RNA was examined by the use of a BioAnalyzer 2100 system with an RNA 6000 nanokit (Agilent, USA). Samples with better RNA integrity number (RIN) values (6.8 to 7.6) (SCN1_day10, SCN3_day10, SN1_day10, and SN3_day10) were selected for mRNA library preparation using a Ribo-Zero rRNA removal kit (Illumina). Fragmentation, cDNA synthesis, A-tailing, adapter ligation, uracil-specific excision reagent enzyme treatment, and PCR enrichment were conducted using TruSeq Stranded LT mRNA Library Prep kit v2 (Illumina). Library profiles were analyzed by the use of a BioAnalyzer 2100 system and a High Sensitivity DNA kit (Agilent, USA), and the average insertion size of libraries ranged from 341 to 348 bp. For sequencing, libraries were run on an Illumina Hiseq 2500 system with a HiSeq TruSeq Rapid Duo cBot sample loading kit and HiSeq Rapid PE cluster kit v2 (paired end; 2 by 151 bp) at the High-Throughput Genomics Core Laboratory, Biodiversity Research Center, Academia Sinica.

### Preprocessing, assembling, quantification, and differential expression analysis of metatranscriptomic data.

The metatranscriptome sequencing provided 30 Gb of output for four samples, generating 64,321,692, 66,102,598, 66,563,202, and 65,014,896 reads from SCN1_day10, SCN3_day10, SN1_day10, and SN3_day10 samples, respectively. The percentages of overall bases that passed the filter (number of quorums sensed [QS] ≥ 30) in each sample ranged from 94.95% to 95.81%. First, the adapters of forward and reverse reads and the low-quality reads (QS < 30) were removed using Trimmomatic v0.36 (71). The forward and reverse reads that remained after trimming were merged using PEAR 0.9.8 (72). SortMeRNA v2.1 (73) was then applied to filter rRNA fragments based on its own prepackaged databases. Reads from all samples were assembled together to generate a single assembly using Trinity v2.5.1 after *in silico* read normalization (74). The numbers of reads that survived after each preprocessing step and the statistics of assembling are listed in Table S2 in the supplemental material. The read counts of assembled transcripts were estimated first by mapping sequencing reads of each sample against the assembled transcripts using Bowtie2 2.3.4.1 (75) and then quantified using featureCounts (76). The edgeR software package (77) was used for analyzing differential gene expression (DGE) between SCN and SN communities with two biological replicates. Transcripts with log_2_ fold change (log_2_ FC) values of ≥2, false-discovery-rate (FDR) values of ≤0.05, and adjusted *P* values of <0.05 were designated DGE transcripts. Volcano plots were drawn using R (https://www.r-project.org).

10.1128/mSystems.00113-18.6 TABLE S2 Read numbers after each step of preprocessing per sample and overall statistics after assembly. Download Table S2, DOCX file, 0.01 MB. Copyright © 2018 Wei et al. 2018 Wei et al. This content is distributed under the terms of the Creative Commons Attribution 4.0 International license.

### Functional annotation for assembled transcripts.

The assembled transcripts were applied to MetaGeneMark v3.25 (78), which predicted 351,342 open reading frames (ORFs). To assign putative functionality between sludge communities treated with and without cholesterol under denitrifying conditions (SCN versus SN), the amino acid sequences of predicted ORFs were functionally annotated against the KEGG Orthology database (79) using GhostKOALA (80).

The KEGG database includes only aerobic steroid degradation pathways. To obtain the functional annotations involved in anaerobic cholesterol degradation, we included protein sequences from newly released complete genomes of Stl. denitrificans (14) into the complete nonredundant protein database of RefSeq release 85 (81, 82); amino acid sequences were aligned against this database using protein aligner DIAMOND v0.9.15 with a cutoff E value of 1e−05 (83). For further gene mining, the hidden Markov model (HMM) search was also applied. Amino acid sequences for proteins involved in ring-A modification (AcmA and AcmB), side chain hydroxylation (C25DH), ring-A degradation (AtcA), and C/D-ring degradation (IpdAB, IpdC and enoyl-CoA hydratase) from anaerobic steroid-degrading Stl. denitrificans, Sdo. denitrificans, and T. terpenica 58Eu were selected for HMM generation (Table S3). These protein sequences were then aligned using MUSCLE 3.8.425 with default settings (84) in Geneious R11.1.2 (85). The alignment outputs were used to build HMMs using HMMER (hmmer.org v3.1b2). Amino acid sequences from our metatranscriptome data set were searched against the generated HMMs using the hmmsearch command with an E value of 1e−5. The candidate genes with scores over 200 were then selected for BLAST searches (blastp) against a nonredundant protein sequence database in NCBI to obtain their putative functions.

10.1128/mSystems.00113-18.7 TABLE S3 The source of sequences used for phylogenetic analysis and HMM searches. Download Table S3, DOCX file, 0.02 MB. Copyright © 2018 Wei et al. 2018 Wei et al. This content is distributed under the terms of the Creative Commons Attribution 4.0 International license.

10.1128/mSystems.00113-18.8 TABLE S4 Two-letter codes used to denote taxa in spider chart. Download Table S4, DOCX file, 0.01 MB. Copyright © 2018 Wei et al. 2018 Wei et al. This content is distributed under the terms of the Creative Commons Attribution 4.0 International license.

### Phylogenetic analysis.

Maximum likelihood trees were constructed to elucidate phylogenetic relationships between steroid-degrading anaerobes (16S rRNA genes), AtcA, and alpha subunits of C25DH (S25dA1–8). Other molybdopterin-containing proteins similar to AtcA (19) and C25DH (86) were selected as outgroups. The sources of the sequences described above for each tree are listed in Table S3. All nucleotide and amino acid sequences were aligned using MUSCLE (84) in MEGA6 (87). Due to the variety in gene lengths of *atcA* and *c25dh* identified in this study, short sequences were discarded for phylogenetic analysis. The best amino acid substitution model for each phylogenetic tree construction was determined by the use of Model Test in MEGA6 (87). The Kimura 2-parameter (K2) plus gamma distribution (G) rates were applied for construction of the maximum likelihood tree of 16S rRNA genes, and the LG model with G rates was applied for AtcA and C25DH phylogenetic analysis. Branch support was determined by bootstrapping 1,000 times. Pairwise analysis of all these 16S rRNA sequences was conducted using Geneious R11 (85).

### Accession number(s).

The raw reads of all 16S amplicons and all metatranscriptome data set were deposited in NCBI Sequence Read Archive (SRA) with BioSample SAMN08848259 under SRA accession no. SRP136909.
