# Supplementary figures and images for: Microbial Functional Responses to Cholesterol Catabolism in Denitrifying Sludge
Source: mSystems. 2018 Oct 30;3(5):e00113-18. doi: 10.1128/mSystems.00113-18 (PMC6208644; doi:10.1128/mSystems.00113-18)

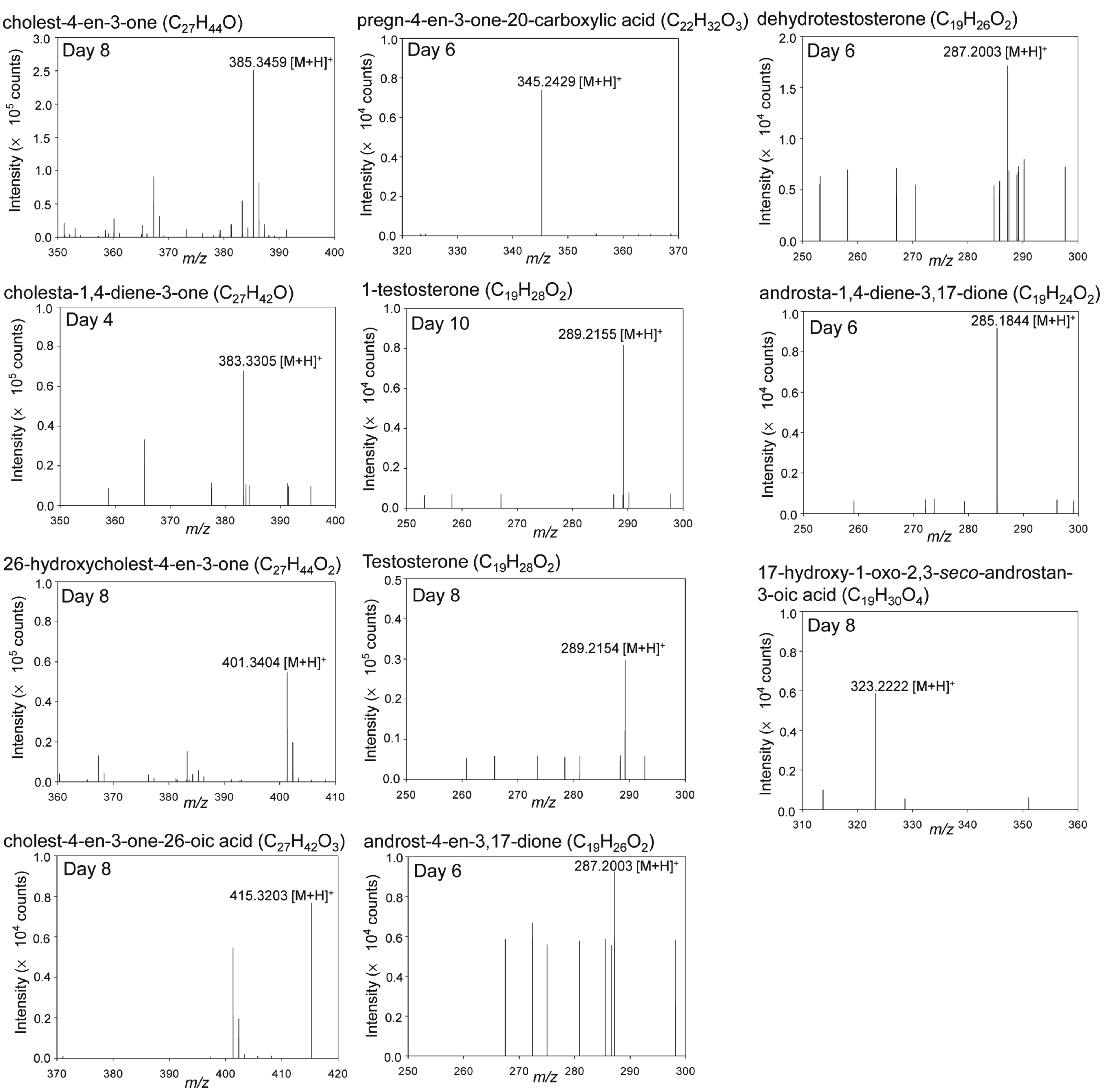

Supplement: FIG S1 [file sys006182282sf1.tif]

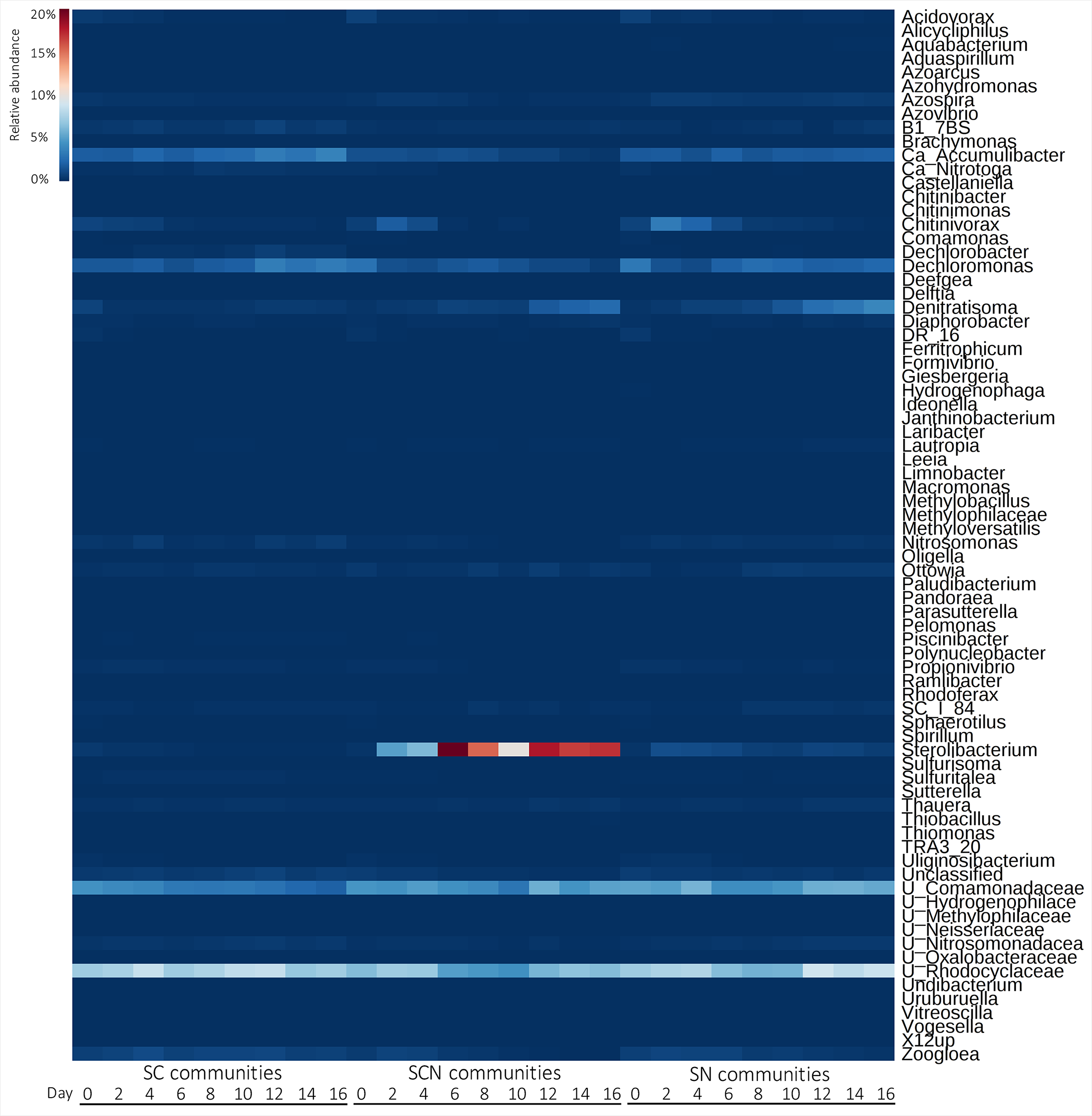

Supplement: FIG S2 [file sys006182282sf2.tif]

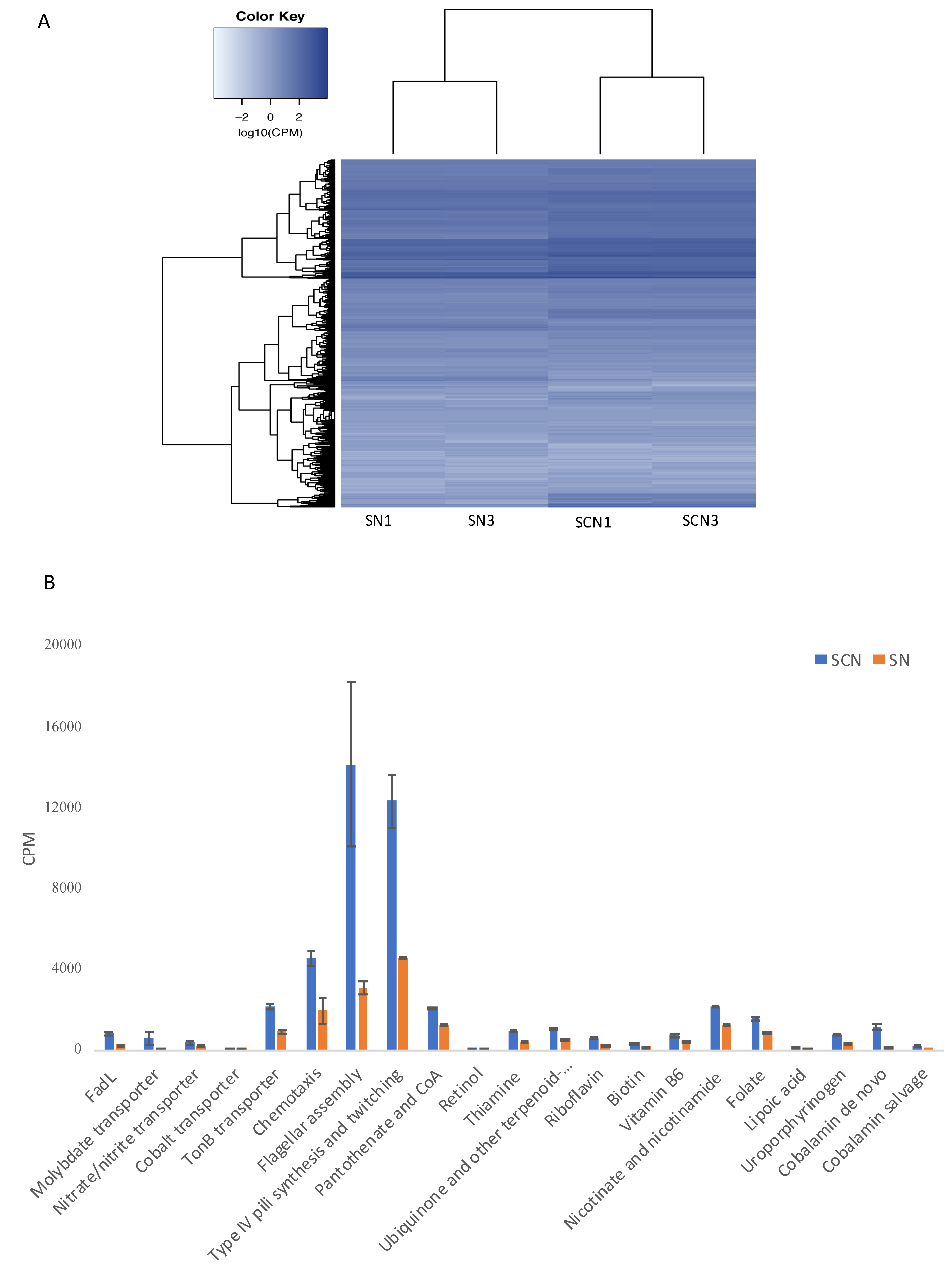

Supplement: FIG S3 [file sys006182282sf3.tif]

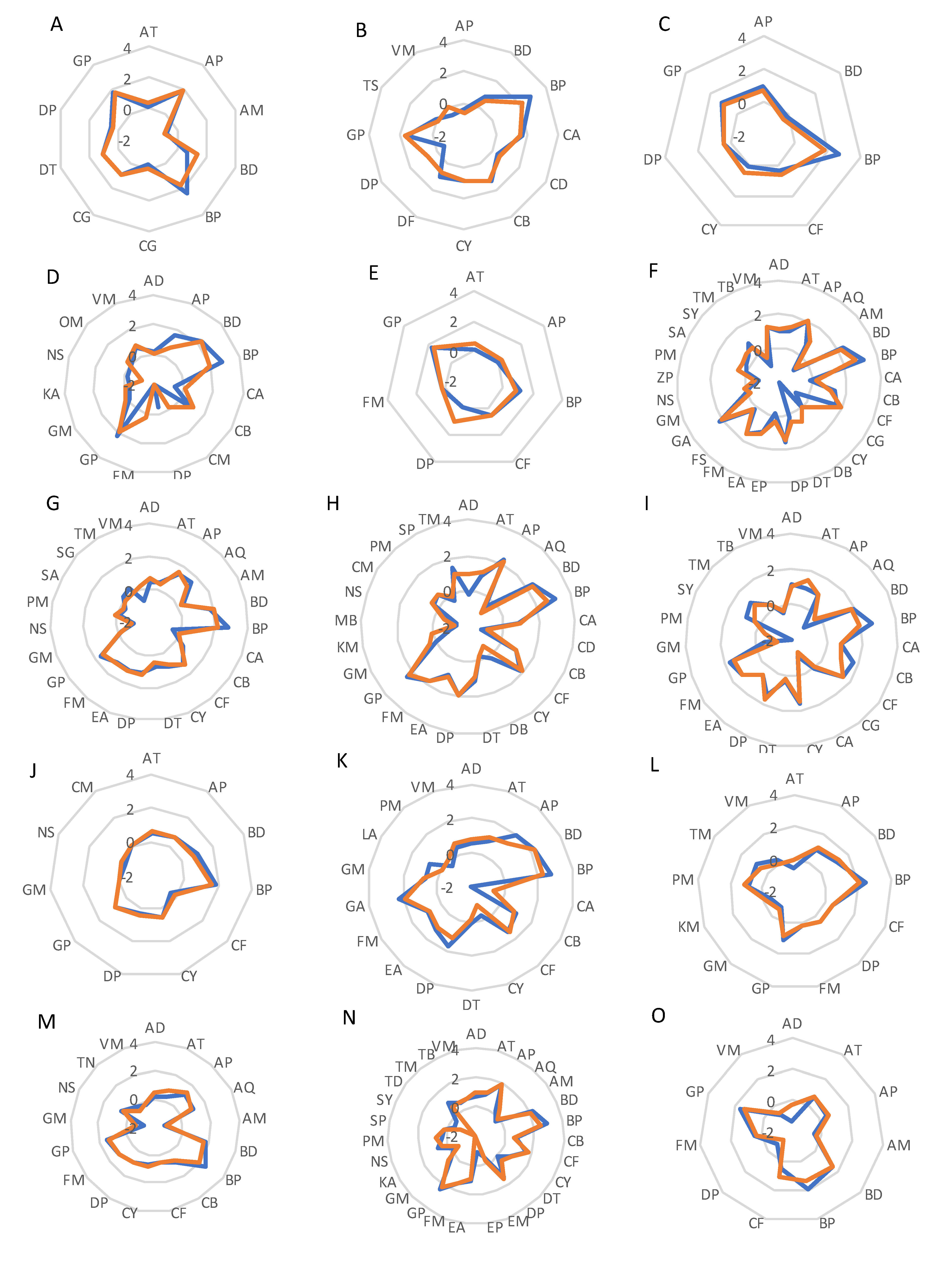

Supplement: FIG S4 [file sys006182282sf4.tif]
