# Supplementary material for: Microbial Functional Responses to Cholesterol Catabolism in Denitrifying Sludge
Source: mSystems. 2018 Oct 30;3(5):e00113-18. doi: 10.1128/mSystems.00113-18 (PMC6208644; doi:10.1128/mSystems.00113-18)
Supplement: TABLE S1 [file sys006182282st1.docx]

| Putative function | Protein | Mean | SD |
| --- | --- | --- | --- |
| Uptake | FadL | 591.66 | 118.77 |
| Ring-A modification | AcmA | 127.89 | 1.45 |
|  | AcmB | 192.88 | 1.01 |
| Side-chain hydroxylation | S25dA1 | 622.24 | 183.39 |
|  | S25dA2 | 65.29 | 7.75 |
|  | S25dA3 | 154.89 | 32.89 |
|  | S25dA4 | 68.39 | 3.07 |
|  | S25dA5 | 355.16 | 97.59 |
|  | S25dA6 | 34.10 | 1.43 |
|  | S25dA7 | 178.68 | 38.89 |
|  | S25dC4 | 1.80 | 0.23 |
|  | S25dD4 | 65.79 | 5.18 |
| Side-chain degradation | C26_ACAD | 23.50 | 8.09 |
|  | C24_ACS | 123.69 | 28.37 |
|  | C24_ACAD | 68.59 | 5.50 |
|  | C22_ACS | 56.29 | 9.01 |
|  | C22_ACAD | 97.09 | 6.93 |
|  | C22_ECH | 72.09 | 6.18 |
|  | C22_ALD | 65.49 | 0.89 |
| A/B-rings degradation | Ketosteroid-Δ4-reductase | 98.49 | 14.65 |
|  | AtcA | 384.36 | 87.63 |
|  | AtcB | 47.10 | 8.40 |
|  | AtcC | 117.09 | 30.99 |
|  | 2,3-SAOA_ACS | 109.58 | 26.52 |
|  | HIP_ACS | 47.00 | 7.85 |
|  | HIP-CoA_ACAD | 115.69 | 7.18 |
|  | CD-rings_ECH | 28.80 | 4.55 |
| C/D-rings degradation  Unknown | D-ring ECH | 41.10 | 2.22 |
|  | IdpA | 36.70 | 1.53 |
|  | IdpB | 62.49 | 4.98 |
|  | IdpC | 35.10 | 4.31 |
|  | CD-rings_TL | 68.10 | 16.47 |
|  | MOODA-CoA_ACAD | 143.39 | 15.18 |
|  | β-oxidation-related | 443.46 | 32.07 |
