# Supplementary material for: Microbial Functional Responses to Cholesterol Catabolism in Denitrifying Sludge
Source: mSystems. 2018 Oct 30;3(5):e00113-18. doi: 10.1128/mSystems.00113-18 (PMC6208644; doi:10.1128/mSystems.00113-18)
Supplement: TABLE S2 [file sys006182282st2.docx]

|  | SCN1_day10 | SCN3_day10 | SN1_day10 | SN3_day10 | Statistics |  |
| --- | --- | --- | --- | --- | --- | --- |
| Raw reads | 64,321,692 | 66,102,598 | 66,563,202 | 65,014,896 |  |  |
| TrimmoaticPE | 306,85,674 | 31,358,551 | 31,720,556 | 32,507,448 |  |  |
| PEAR | 28,640,608 | 29,494,911 | 29,958,554 | 28,496,418 |  |  |
| SortMeRNA (rRNA) | 1,687,234 | 1,516,502 | 1,317,669 | 970,017 |  |  |
| SortMeRNA (mRNA)* | 26,953,374 | 27,978,409 | 28,640,885 | 27,526,401 |  |  |
| Total Trinity “genes” |  |  |  |  | 183,057 |  |
| Total Trinity transcripts |  |  |  |  | 215,266 |  |
| Contig N50 |  |  |  |  | 1278 |  |
| Median contig length |  |  |  |  | 632 |  |
| Average contig |  |  |  |  | 948.23 |  |
| Total assembled bases |  |  |  |  | 204,123,548 |  |
| Total GC% |  |  |  |  | 59.88 |  |

*Reads that applied to Trinity assembly and abundance estimation using Bowtie2
