# Supplementary material for: Microbial Functional Responses to Cholesterol Catabolism in Denitrifying Sludge
Source: mSystems. 2018 Oct 30;3(5):e00113-18. doi: 10.1128/mSystems.00113-18 (PMC6208644; doi:10.1128/mSystems.00113-18)
Supplement: TABLE S3 [file sys006182282st3.docx]

| Gene/enzyme | Taxonomy | Accession No. | Analysis |
| --- | --- | --- | --- |
| *Sterolibacterium denitrificans* DSMZ13999 16S rRNA | Betaproteobacteria; Nitrosomonadales; Sterolibacteriaceae; Sterolibacterium | NR_025450.1  NCBI | Phylogenetic analysis |
| *Denitratisoma oestradiolicum* DSMZ18526 16S rRNA | Betaproteobacteria; Nitrosomonadales; Sterolibacteriaceae; Denitratisoma | NR_043249.1  NCBI |  |
| *Thauera terpenica* 58Eu  16S rRNA | Betaproteobacteria; Rhodocyclales;  Zoogloeaceae; *Thauera* | NZ_ATJV01000095  NCBI |  |
| *Steroidobacter denitrificans* DSMZ18526 16S rRNA | Gammaproteobacteria; Nevskiales; Sinobacteraceae; Steroidobacter | NZ_CP011971  NCBI |  |
| *Thauera* sp. GDN1  16S rRNA | Betaproteobacteria; Rhodocyclales;  Zoogloeaceae; *Thauera* | (1) |  |
| Michael hydratase/alcohol dehydrogenase | Betaproteobacteria;  *Alicycliphilus denitrificans* DSM 14773 | ADV16271.1  NCBI |  |
| Carbon monoxide dehydrogenase | Chloroflexi;  *Thermomicrobium roseum* DSM5159 | ACM04661  NCBI |  |
| Caffeine dehydrogenase | Gammaproteobacteria;  *Pseudomonas* sp. CBB1 | D7REY3  Swis-Port |  |
| Carbon monoxide dehydrogenase | Alphaproteobacteria;  *Oligotrophia carboxidovorans* DSM1227 | P19919  Swiss-Port |  |
|  |  |  |  |
| 4-hydroxybenzoyl-CoA-reductase | Betaproteobacteria;  *Thauera aromatica* | O33819  Swiss-port |  |
|  |  |  |  |
| Ethylbenzene dehydrogenase  (alpha subunit) | Betaproteobacteria;  *Azoarcus toluclasticus* ATCC700605 | WP_018992917.1  NCBI |  |
|  |  |  |  |
| AcmA | *Stl. denitrificans* DSM13999 | SMB27870.1 | HMMs |
|  |  | SMB27339.1 |  |
|  |  | SMB21939.1 |  |
|  |  | SMB21130.1 |  |
| AcmB | *Stl. denitrificans* DSM13999 | SMB21450.1 |  |
|  |  | SMB22076.1 |  |
|  |  | SMB21450.1 |  |
| C25DH_alpha subunit | *Stl. denitrificans* DSM13999 | SMB29354.1 |  |
|  |  | SMB29206.1 |  |
|  |  | SMB27756.1 |  |
|  |  | SMB26755.1 |  |
|  |  | SMB22287.1 |  |
|  |  | SMB22299.1 |  |
|  |  | SMB23158.1 |  |
|  |  | SMB25046.1 |  |
|  | *Sdo. denitrificans* DSM18526 | AMN47566.1 |  |
|  | *T. trepenica* 58Eu | EPZ14817.1 |  |
| AtcA | *Stl. denitrificans* DSM13999 | SMB21166.1 |  |
|  | *Sdo. denitrificans* DSM 18526 | AMN46163.1 |  |
|  | *T. trepenica* 58Eu | EPZ16113.1 |  |
| IpdA | *Stl. denitrificans* DSM13999 | SMB21413.1 |  |
|  | *Sdo. denitrificans* DSM 18526 | WP_016491273.1 |  |
|  | *T. trepenica* 58Eu | WP_021250110.1 |  |
| IpdB | *Stl. denitrificans* DSM13999 | SMB21414.1 |  |
|  | *Sdo. denitrificans* DSM 18526 | WP_066917840.1 |  |
|  | *T. trepenica* 58Eu | WP_021250109.1 |  |
| IpdC | *Stl. denitrificans* DSM13999 | SMB21419.1 |  |
|  | *Sdo. denitrificans* DSM 18526 | WP_066917843.1 |  |
|  | *T. trepenica* 58Eu | WP_021250107.1 |  |
| Enoyl-CoA hydratase | *Stl. denitrificans* DSM13999 | SMB21421.1 |  |
|  | *Sdo. denitrificans* DSM 18526 | WP_066917844.1 |  |
|  | *T. trepenica* 58Eu | WP_021250108.1 |  |

1. Shih C-J, Chen Y-L, Wang C-H, Wei ST-S, Lin I-T, Ismail WA, Chiang Y-R. 2017. Biochemical Mechanisms and Microorganisms Involved in Anaerobic Testosterone Metabolism in Estuarine Sediments. Front Microbiol 8:1520.
