# Supplementary material for: Microbial Functional Responses to Cholesterol Catabolism in Denitrifying Sludge
Source: mSystems. 2018 Oct 30;3(5):e00113-18. doi: 10.1128/mSystems.00113-18 (PMC6208644; doi:10.1128/mSystems.00113-18)
Supplement: TABLE S4 [file sys006182282st4.docx]

| **Code** | **Taxon** | **Code** | **Taxon** |
| --- | --- | --- | --- |
| AD | Acidobacteria | LA | Lokiarchaeota |
| AP | Alphaproteobacteria | NP | Nitrospirae |
| AM | Armatimonadetes | PM | Planctomycetes |
| AQ | Aquificae | SP | Spirochaetes |
| AT | Actinobacteria | SB | Saccharibacteria |
| BD | Bacteroidetes | SY | Synergistetes |
| BP | Betaproteobacteria | TA | Thaumarchaeota |
| CB | Chlorobi | TB | Thermobaculum |
| CA | Calditrichaeota | TD | Thermodesulfobacteria |
| CD | Chlamydiae | TM | Thermotogae |
| CF | Chloroflexi | VM | Verrucomicrobia |
| CL | Cloacimonetes | ZP | Zetaproteobacteria |
| CM | Candidatus Methylomirabilis |  |  |
| CA | Crenarchaeota |  |  |
| CY | Cyanobacteria |  |  |
| DB | Deferribacteres |  |  |
| DP | Deltaproteobacteria |  |  |
| DT | Deinococcus-Thermus |  |  |
| DG | Dictyoglomi |  |  |
| EA | Euryarchaeote |  |  |
| EM | Elusimicrobia |  |  |
| EP | Epsilonproteobacteria |  |  |
| EA | Euryarchaea |  |  |
| FB | Fibrobacteres |  |  |
| FM | Firmicutes |  |  |
| FB | Fusobacteria |  |  |
| GM | Gemmatimonadetes |  |  |
| GP | Gammaproteobacteria |  |  |
| KM | Kiritimatiellaeota |  |  |
